# Comparative study of the conservative and surgical treatment of spontaneous cerebellar hemorrhage

**DOI:** 10.3389/fneur.2025.1678837

**Published:** 2025-12-09

**Authors:** Chaozhen Yang, Lei Huang, Ping Xu, Zhiyuan Zhang, Wei Che, Siying Ren, Guofeng Wu, Likun Wang

**Affiliations:** 1 Emergency Department, The Affiliated Hospital of Guizhou Medical University, Guiyang, Guizhou Province, China; 2 Department of Emergency, Guizhou Hospital of The First Affliated Hospital, Sun Yat-sen University, Guiyang, China; 3 Endocrinology and Metabolism Department of Guizhou Medical University Affiliated Hospital, Guiyang, China

**Keywords:** spontaneous cerebellar hemorrhage, conservative treatment, surgical treatment, prognosis, intracerebral hemorrhage

## Abstract

**Background and purpose:**

The mortality rate of spontaneous cerebellar hemorrhage (SCH) is extremely high. Currently, only surgical treatment (ST) and conservative treatment (CT) methods are available; however, the indications for the treatment of SCH are not yet clear. In this study, we compared the outcomes of conservative and surgical treatment methods for patients with SCH and a hematoma volume of >10 mL.

**Methods:**

We retrospectively included patients with SCH who were treated in the Emergency Department of the Affiliated Hospital of Guizhou Medical University, the Neurosurgery Department of the Affiliated Jinyang Hospital of Guizhou Medical University, and the Neurosurgery Department of the Second Affiliated Hospital of Guizhou Medical University from April 2014 to January 2024. Patients were divided into CT group and ST group using a 1:2 stratified matching method based on hematoma volume and diameter. We collected baseline clinical characteristics of patients, including age, blood pressure, imaging data, complications, and prognosis, and conducted univariate analysis. After excluding factors with collinearity effects through collinearity diagnosis, we used a binary logistic regression model to analyze the independent correlation between good and poor prognosis.

**Results:**

Based on the inclusion criteria, 98 patients with SCH were screened, comprising 41 patients in the CT group and 57 patients in the ST group. Univariate analysis showed that the ST group had a higher proportion of patients with good prognosis at 1 and 3 months [41(71.9%) vs. 19(46.3%), *p* = 0.010], [34(59.6%) vs. 14(34.1%), *p* = 0.013], and a lower mortality rate than the CT group [10(17.9%) vs. 15(39.5%), *p* = 0.020]. SCH is further divided into a 1-month good prognosis group and a poor prognosis group, and a 3-month good prognosis group and a poor prognosis group. After excluding factors through collinearity diagnosis, the results of multivariate binary logistic regression analysis showed that surgical treatment had better 1- and 3-month prognosis than conservative treatment in SCH patients (OR: 4.898, 95% CI: 1.559–15.388, *p* = 0.007, OR: 3.965, 95% CI: 1.429–11.004, *p* = 0.008).

**Conclusion:**

When the bleeding volume of SCH patients is greater than 10 mL, surgery is an independent predictor of good short-term prognosis.

## 1 Introduction

SCH accounts for 10% of all intracranial hemorrhages but has very high mortality and disability rates, with mortality rates as high as 50% (1). Hypertension is the most common cause of SCH (2), with SCH often located in the dentate nucleus. Hematomas compress the cerebellar peduncle or fourth ventricle, leading to life-threatening hernias of the foramen magnum. The posterior cerebellar fossa is of particular clinical significance in the context of SCH, as its distinctive anatomical structure has been shown to portend unfavorable prognoses for patients with this condition. This particular aspect of the posterior fossa may even be associated with an elevated risk of mortality (3).

In 2022, the American Stroke Association proposed that hematoma evacuation should be performed when the SCH bleeding volume reaches 15 mL (4), with a significant correlation shown between survival rate and hematoma evacuation (5). Studies have found that regardless of the age of patients and the size of the hematoma, patients with SCH should undergo suboccipital decompression to remove the hematoma to facilitate earlier neurological recovery (6). A suboccipital craniotomy is more significant than conservative treatment for hematoma removal and has a better neurological prognosis (7). ST mainly includes craniotomy, decompressive craniectomy, hematoma evacuation, and stereotactic minimally invasive intracranial hematoma evacuation. The removal of hematomas by craniotomy with bone flap removal is significant (7) and has become one of the most commonly used operations in SCH (4). In addition, minimally invasive surgery to remove SCH is both safe and effective, with the ability to improve neurological function and reduce mortality (8, 9). In supratentorial cerebral hemorrhage, minimally invasive intracranial hematoma removal can effectively reduce the mortality of patients (4). The French Society of Neurosurgery and the French Society of Vascular Neurology proposed that when the SCH hematoma volume is >15 cm^3^ and the Glasgow Coma Scale is <10, the hematoma should be surgically cleared to improve patient prognosis (10).

Additionally, CT usually brings good results for patients with SCH (11). Some studies have demonstrated no significant difference in modified Rankin Scale (mRS) between ST and CT within 3 months, concluding that surgical removal of blood swelling was unrelated to the improvement in functional outcomes (12). In a retrospective study of 57 patients with SCH conducted in 2020, the poor prognoses of the CT group and the ST group were 36 and 72%, respectively (13). ST may be beneficial for patients with severe SCH whose intracerebral hemorrhage (ICH) score is >3, whereas CT seems reasonable for patients with a lower intracerebral hemorrhage score (14).

Prognoses for patients with benign SCH are generally favorable (15). However, when the volume of hemorrhage reaches 10 mL, patients may already exhibit poor neurological changes. At this point, the optimal course of action is a matter of debate. Some patients advocate a conservative approach, whereas others favor surgical intervention. A previous study proved that the prognosis of the ST group is better than that of the CT group when the hematoma volume is >10 mL. It should be noted, however, that this was a small-sample, single-center study (16). Our study included SCH with a hematoma volume greater than 10 mL, and the threshold was set based on the unique anatomical structure of the posterior cranial fossa - even small hematomas may have significant occupying effects on the brainstem or cause obstructive hydrocephalus. Although there are guidelines for large amounts of SCH (e.g., > 15 mL), there is a lack of strong evidence for patients with large amounts of moderate volume hematoma (e.g., > 10 mL), which creates a clear gray area in clinical decision-making. This study aims to adopt more proactive intervention strategies for this key subgroup of patients.

## 2 Methods and procedures

This retrospective clinical observational study complied with the basic principles of the World Medical Association’s Declaration of Helsinki. Clinical data were analyzed in accordance with the policy requirements of the Ethics Committee of Guizhou Medical University (17) (Approval No. 2019/114, 2023 Lunshen No. 404). The trial is registered at ClinicalTrials.gov (NCT05548530).

### 2.1 Study design

A total of 112 eligible patients with SCH and related clinical data from April 2014 to January 2024 were included in this study, including those from the Emergency Department of the Affiliated Hospital of Guizhou Medical University, the Affiliated Jinyang Hospital of Guizhou Medical University, and the Second Affiliated Hospital of Guizhou Medical University. All patients admitted to the emergency Neurology or Neurosurgery Department underwent neurological evaluation by clinical physicians in each department. After a comprehensive review of the patients’ clinical status and records, all patients reached a consensus on the treatment methods.

**Figure 1:**
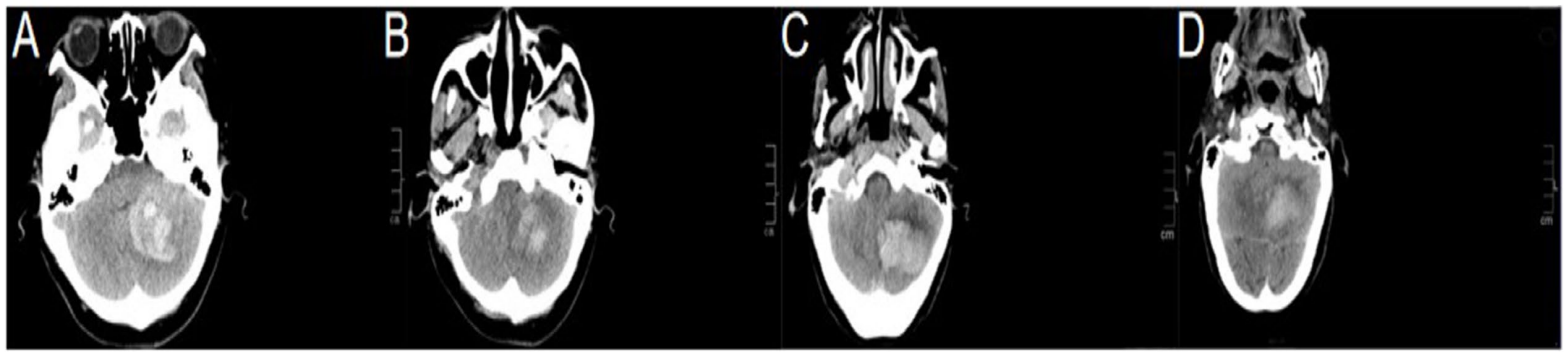
**(A–D)** All brain computed tomography scan results before and after conservative treatment.

**Figure 2:**
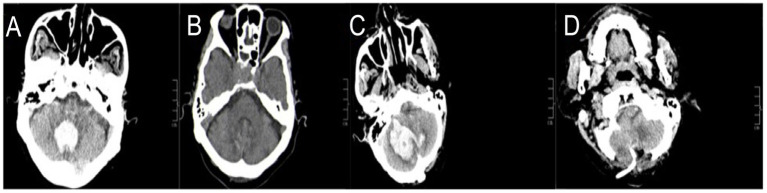
**(A–D)** The results of brain computed tomography scan before and after surgical treatment.

The inclusion criteria were as follows: (1) age ≥ 18 years; (2) baseline brain computed tomography scan performed within 6 h after the onset of symptoms, and the bleeding site should be clearly in the cerebellum, with a bleeding volume of >10 mL and/or accompanied by brainstem compression, fourth ventricle obstruction, cerebral pool obstruction, hydrocephalus, and deterioration of consciousness; and (3) patients who underwent CT (Figures 1A–D), stereotactic minimally invasive intracranial hematoma removal surgery (SMIS) (Figures 2A,B), or craniotomy with bone flap decompression hematoma removal surgery (CBDS) (Figures 2C,D).

Our data originated from three distinct regions, all directly affiliated with the Guizhou Medical University Hospital System, implying their adherence to identical clinical treatment pathways, surgical protocols, and quality control standards as established and led by the University. Their medical teams share common origins and undergo uniform training and evaluations, ensuring a high degree of homogeneity in diagnostic and therapeutic standards.

The exclusion criteria were as follows: (1) secondary SCH caused by traumatic brain injury, cavernous hemangioma, intracranial arteriovenous malformation, moyamoya disease, intracranial tumor, cerebral infarction, and intracranial aneurysm, among others; (2) primary intraventricular hemorrhage; and (3) incomplete or missing follow-up information. All patients who met the criteria for ST were included in the surgical group, while patients in the CT group were matched 1:2 with ST using a stratified matching method based on hematoma volume and diameter.

### 2.2 Treatment allocation criteria

The decision to perform surgery or to pursue conservative management was made on a case-by-case basis by our institutional multidisciplinary neurovascular team. In general, surgical evacuation was strongly considered in the presence of any of the following: (1) Hematoma volume> 15 mL Or hematoma diameter greater than 3 cm (4), (2) evidence of brainstem compression on computed tomography, (3) progressive neurological deterioration, (4) obstructive hydrocephalus, (5) Obtain consent from family members for surgery and sign relevant informed consent forms (10). On the contrary, actively adopt CT.

### 2.3 Data acquisition

**Figure 3:**
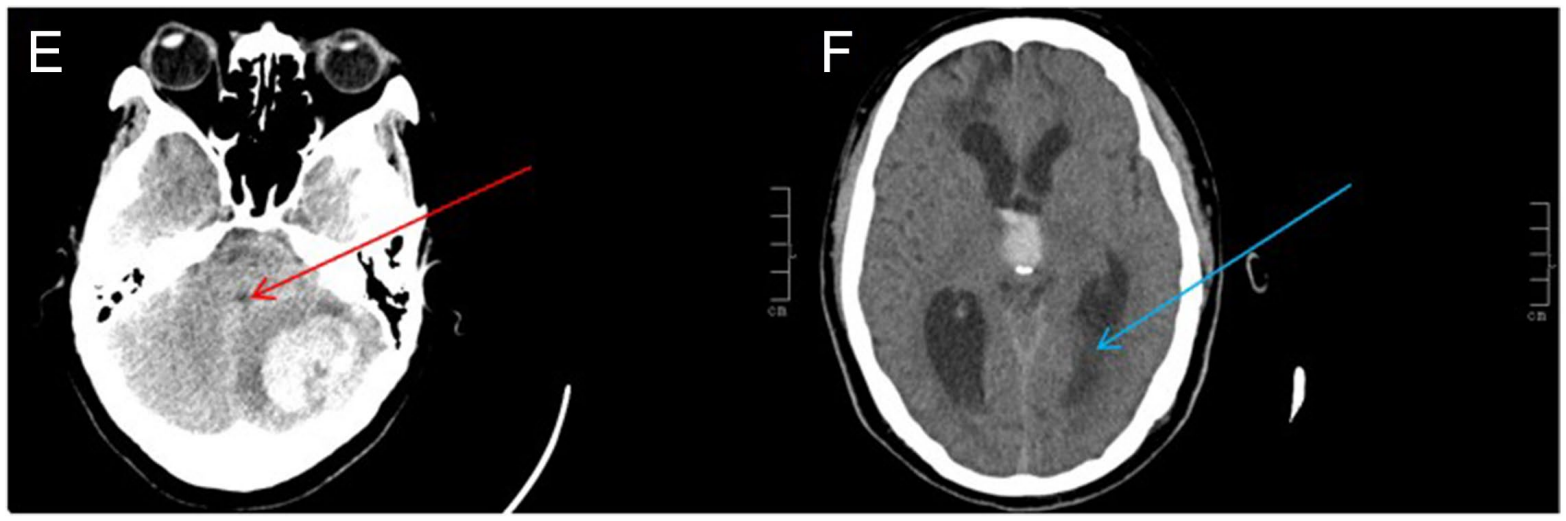
Tight posterior fossa:(E, red arrow) fourth ventricle compression, and/or (F, blue arrow) dilated ventricle after obstructive hydrocephalus (3, 20, 38).

It is imperative to collect patients’ sex, age, alcohol consumption history, smoking history, Glasgow Coma Scale (GCS) upon admission, and National Institutes of Health Stroke Scale (NIHSS) score to clarify the baseline hematoma volume, presence of tight posterior fossa (Figure 3), functional outcomes defined by the modified Rankin Scale (mRS) and 30-d prognosis and 3-month prognosis (18), and related complications. The patient or family member is required to sign an informed consent form.

### 2.4 Primary and secondary outcomes

We analyzed the prognostic accuracy of surgical treatment (ST) in patients with SCH using binary logistic regression. The primary outcomes of the analysis were prognosis the patients 30-d and 3-month later, while the secondary outcomes included the occurrence of minor complications related to the procedure. We divided the patients into two groups: ST and CT groups or good and poor prognosis groups. Surgical treatment (ST) was defined as follows: stereotactic minimally invasive intracontinental hematoma removal surgery (SMIS) with standard protocol approvals and patient consent (Figures 2A,B) (17, 19) or craniotomy with bone flap decompression and hematoma removal surgery (CBDS) (Figures 2C,D) (6, 7).

### 2.5 Imaging analysis

The hematoma volume was measured using the ABC/2 method, where A is the greatest hemorrhage diameter on brain computed tomography scan, B is the diameter perpendicular to A, and C is the approximate number of computed tomography scan slices with hemorrhage multiplied by the slice thickness (20, 21).

### 2.6 Prognostic measurement criteria

The mRS score was used to assess prognosis. The mRS score of 0–3 is defined as “good prognosis,” and 4–6 is defined as “poor prognosis” (22). The setting of this boundary point refers to the research conventions in the field of cerebellar hemorrhage: mRS score 0–2 represents functional independence, while mRS score 3 represents the ability to walk independently despite moderate disability (12). This has important clinical significance for the recovery of balance and coordination function after cerebellar hemorrhage. The selection of 1-month and 3-month time points aims to evaluate the early effects of treatment strategies, during which the patient’s neurological function typically tends to stabilize and can effectively reflect differences in different intervention measures.

### 2.7 Bias control measures

The following methods were used to minimize potential sources of bias. According to the hematoma volume and maximum diameter, a 1:2 stratified matching method is used to minimize selection bias. Our data originated from three distinct regions, all directly affiliated with the Guizhou Medical University Hospital System, implying their adherence to identical clinical treatment pathways, surgical protocols, and quality control standards as established and led by the University. Their medical teams share common origins and undergo uniform training and evaluations, ensuring a high degree of homogeneity in diagnostic and therapeutic standards. Perform collinear diagnosis and binary logistic regression analysis to control for confounding factors such as baseline clinical features.

### 2.8 Statistical analysis

The study was to use the statistical software R 4.4.2 and SPSS 27.0. To reduce selection bias between treatment groups, we first performed propensity score matching. Patients in the conservative group were matched with the surgical group in a 1:2 ratio using stratified matching based on hematoma volume and diameter. Secondly, in the matched queue, two sets of univariate analyses were conducted: (1) comparing baseline characteristics between the surgical and conservative treatment groups (ST vs. CT) to describe inter group differences (Table 1); (2) Compare the characteristics of good and poor prognosis groups to screen candidate variables for multivariate models (*p* < 0.05) (Table 2). in the queue, In the case of measurement data with a normal distribution, the mean ± standard deviation (X ± S) was used to represent the data. In contrast, for measurement data that did not follow a normal distribution, the median (M, Q1, Q3) was used. The differences between the groups were compared using a t-test, while the count data were compared using a chi-squared test, with the results expressed as percentages (n, %). Perform collinearity diagnosis on variables with *p* < 0.05 in univariate analysis of good and poor prognosis to evaluate their collinearity strength, and then exclude collinearity influencing factors with VIF > 3. Finally Multivariate binary logistic regression analysis of relevant factors, with a *p* < 0.05 indicating statistical significance (23).

**Table 1:** Baseline comparison of the two treatment methods.

Variables	Total (*n* = 98)	CT group (*n* = 41)	ST group (*n* = 57)	*t/Z/X^2^*	*P*
Sex (male, %)	61(62.2%)	25(61.0%)	36(63.2%)	0.048	0.826
Age (year, ▲)	65.18 ± 12.78	67.32 ± 15.05	63.73 ± 10.88	3.131	0.194
Hospitalization days (day, IQR)	16.00(9.00, 23.25)	16.00(4.75, 21.25)	17.00(11.00, 25.00)	−1.546	0.122
Smoking (n, %)	33(33.7%)	11(26.8%)	22(38.6%)	1.478	0.224
Alcohol consumption (n, %)	26(26.5%)	10(24.4%)	16(28.1%)	0.166	0.684
Hypertension (n, %)	76(77.6%)	27(65.9%)	49(86.0%)	5.540	0.019*
Diabetes mellitus (n, %)	10(10.3%)	5(12.2%)	5(8.9%)	0.273	0.601
Systolic blood pressure (mmHg, IQR)	170.50(152.25,186.25)	164.00(147.75,180.50)	173.50(157.00,194.75)	−1.639	0.101
Diastolic blood pressure (mmHg, ▲)	100.00(88.75,111.25)	99.00(87.75,110.25)	100.00(89.00,112.75)	−0.256	0.798
NIHSS (divide, IQR)	6.00(3.00, 21.50)	5.00(2.00, 23.00)	6.00(3.00, 21.00)	−0.191	0.848
GCS (divide, IQR)	13.00(9.00, 14.00)	13.00(7.00, 15.00)	12.00(9.00, 14.00)	−0.124	0.902
Hematoma volume (mL, IQR)	15.53(12.75, 18.27)	14.82(11.74, 19.12)	15.77(13.29, 18.09)	−0.630	0.529
Maximum diameter of hematoma (cm, IQR)	3.96 ± 0.68	3.93 ± 0.67	3.98 ± 0.69	0.058	0.487
Break into ventricle (n, %)	68(69.4%)	25(61.0%)	43(75.4%)	2.348	0.125
Obstructive hydrocephalus (n, %)	36(36.7%)	14(34.1%)	22(38.6%)	0.203	0.652
Hematoma enlargement (n, %)	12(12.2%)	4(9.8%)	8(14.0%)	0.406	0.524
Cerebral hernia (n, %)	9(9.2%)	4(9.8%)	5(8.8%)	0.028	0.868
Secondary epilepsy (n, %)	1(1.1%)	0(0.0%)	1(1.8%)	0.668	0.414
Pulmonary complications (n, %)	32(34.4%)	12(32.4%)	20(35.7%)	0.106	0.744
Cardiac complications (n, %)	6(6.5%)	4(10.8%)	2(3.6%)	1.935	0.164
Death (n, %)	25(26.6%)	15(39.5%)	10(17.9%)	5.419	0.020*
Tight posterior fossa (n, %)	34(34.7%)	17(41.5%)	17(29.8%)	1.426	0.232
Platelet (×10^9^/L, %)	189.18 ± 61.75	192.33 ± 60.81	187.04 ± 62.84	0.061	0.468
Neutrophils (×10^9^/L, %)	87.55(80.90, 90.70)	86.95(80.23, 92.15)	88.05(81.50, 90.50)	−0.018	0.985
Lymphocyte (×10^9^/L, %)	7.60(5.38, 12.00)	7.90(4.40,12.23)	7.60(4.40, 12.23)	−0.557	0.578
Leukocyte (×10^9^/L, %)	11.51(9.73, 14.58)	10.43(8.34, 12.68)	12.89(10.39, 16.10)	−2.668	0.008*
Neutrophils/lymphocytes (%)	11.43(6.41, 17.24)	9.95(6.15, 20.90)	11.54(7.00, 15.72)	−0.315	0.753
Platelets/lymphocytes (%)	23.75(15.22, 35.96)	23.30(15.61, 36.80)	24.85(13.10, 35.46)	−0.491	0.623
Percentage of monocytes (×10^9^/L, %)	4.05(3.00, 0.5.73)	4.20(2.85, 5.80)	4.00(3.03, 5.75)	−0.260	0.795
30-d prognosis (n, %)	60(61.2%)	19(46.3%)	41(71.9%)	6.577	0.010*
3-month prognosis (n, %)	48(49.0%)	14(34.1%)	34(59.6%)	6.207	0.013*

CT, Computed tomography; GCS, Glasgow Coma Scale; IQR, Interquartile range; mRS, Modified Rankin Scale; NIHSS, National Institutes of Health Stroke Scale; and ▲ = ^−^x ± s. *Significant variables at the *P* < 0.05 level.

**Table 2:** 30-d prognosis and 3-month univariate analysis of good and poor prognoses.

Variables total (*n* = 98)		30-d prognosis				3-month prognosis			
		Good prognosis (*n* = 60)	Poor prognosis (*n* = 38)	*t/Z/X^2^*	*P*	Good prognosis (*n* = 48)	Poor prognosis (*n* = 50)	*t/Z/X^2^*	*P*
Sex (male, %)	61(62.2%)	41(68.3%)	20(52.6%)	2.441	0.118	34(70.8%)	27(54.0%)	2.953	0.086
Age (year, ▲)	65.18 ± 12.78	64.24 ± 12.69	66.69 ± 12.94	0.060	0.402	63.61 ± 12.64	66.69 ± 12.86	0.076	0.259
Hospitalization days (day, IQR)	16.00(9.00, 23.25)	17.00(12.75, 26.00)	12.00(4.00, 20.00)	−2.616	0.009*	17.00(12.00, 24.25)	15.50(4.25, 22.50)	−1.341	0.180
Smoking (n, %)	33(33.7%)	22(36.7%)	11(28.9%)	0.621	0.431	17(35.4%)	16(32.0%)	0.128	0.721
Alcohol consumption (n, %)	26(26.5%)	18(30.0%)	8(21.1%)	0.956	0.328	15(31.3%)	11(22.0%)	1.075	0.300
Hypertension (n, %)	76(77.6%)	50(83.3%)	26(68.4%)	2.972	0.085	40(83.3%)	36(72.0%)	1.807	0.179
Diabetes mellitus (n, %)	10(10.3%)	7(11.9%)	3(7.9%)	0.394	0.530	5(10.6%)	5(10.0%)	0.011	0.918
Systolic blood pressure (mmHg, IQR)	170.50(152.25,186.25)	171.50(158.25,186.25)	167.00(139.25,192.75)	−1.119	0.263	171.50(158.25,193.25)	169.00(148.00,185.75)	−1.024	0.306
Diastolic blood pressure (mmHg, ▲)	100.00(88.75,111.25)	100.00(90.75,112.00)	99.00 (81.00, 109.75)	−1.163	0.245	99.50(89.75,109.50)	100.00(86.25,112.00)	−0.434	0.665
NIHSS (divide, IQR)	6.00(3.00,21.50)	5.00(1.75,8.00)	18.00(5.25,35.00)	−4.416	0.001*	5.00(1.00,8.25)	14.00(4.00,35.00)	−3.873	0.001*
GCS (divide, IQR)	13.00(9.00,14.00)	13.50(12.00,15.00)	9.00(6.00,13.00)	−4.505	0.001*	14.00(12.00,15.00)	11.50(6.00,13.00)	−4.129	0.001*
Hematoma volume (mL, IQR)	15.53(12.75,18.27)	15.86(12.68,18.37)	14.91(12.80,18.24)	−0.230	0.818	15.77(12.29,18.46)	15.31(13.06,18.06)	−0.217	0.828
Maximum diameter of hematoma (cm, IQR)	3.96 ± 0.68	3.96 ± 0.63	3.97 ± 0.76	1.574	0.989	3.99 ± 0.62	3.94 ± 0.74	0.504	0.713
Break into ventricle (n, %)	68(69.4%)	39(65.0%)	29(76.3%)	1.402	0.236	31(64.6%)	37(74.0%)	1.022	0.312
Obstructive hydrocephalus (n, %)	36(36.7%)	18(30.0%)	18(47.4%)	3.020	0.082	13(27.1%)	23(46.0%)	3.771	0.052
Hematoma enlargement (n, %)	12(12.2%)	5(8.3%)	7(18.4%)	2.203	0.138	4(8.3%)	8(16.0%)	1.340	0.247
Cerebral hernia (n, %)	9(9.2%)	2(3.3%)	7(18.4%)	6.350	0.012*	2(4.2%)	7(14.0%)	2.839	0.092
Secondary epilepsy (n, %)	1(1.1%)	0(0.0%)	1(2.9%)	1.754	0.185	0(0.0%)	1(2.2%)	1.033	0.309
Pulmonary complications (n, %)	32(34.4%)	16(27.1%)	16(74.1%)	3.800	0.051	14(29.8%)	18(39.1%)	0.899	0.343
Cardiac complications (n, %)	6(6.5%)	2(3.4%)	4(11.8%)	2.507	0.113	2(4.3%)	4(8.7%)	0.759	0.384
Death (n, %)	25(26.6%)	5(8.5%)	20(57.1%)	26.653	0.001*	3(6.4%)	22(46.8%)	19.672	0.001*
Tight posterior fossa (n, %)	34(34.7%)	15(25.0%)	19(50.0%)	6.148	0.011*	12(25.0%)	22(44.0%)	3.902	0.048*
Platelet (×10^9^/L, %)	189.18 ± 61.75	199.83 ± 60.48	172.02 ± 60.69	0.006	0.037*	202.80 ± 60.36	176.12 ± 60.83	0.004	0.035*
Neutrophils (×10^9^/L, %)	87.55(80.90, 90.70)	87.75(82.57, 90.50)	87.45(77.33, 91.98)	−0.301	0.764	87.30(80.90, 90.43)	87.90(80.75, 92.03)	−0.855	0.392
Lymphocyte (×10^9^/L, %)	7.60(5.38, 12.00)	7.75(5.70, 10.48)	6.90(4.48, 12.85)	−0.524	0.601	8.10(6.85, 12.30)	6.45(4.40, 11.98)	−1.894	0.058
Leukocyte (×10^9^/L, %)	11.51(9.73, 14.58)	11.72(9.92, 15.59)	11.06(8.74, 13.39)	−1.445	0.149	11.51(9.91, 14.91)	11.49(9.68, 14.39)	−0.541	0.588
Neutrophils/lymphocytes (%)	11.43(6.41, 17.24)	11.30(7.95, 15.40)	12.37(5.45, 20.56)	−0.204	0.838	11.05(6.87, 13.04)	13.67(6.25, 20.72)	−1.627	0.104
Platelets/lymphocytes (%)	23.75(15.22, 35.96)	23.37(16.05, 36.80)	24.51(12.03, 35.46)	−0.498	0.619	22.74(15.75, 31.60)	25.95(12.53, 37.99)	−0.722	0.471
Percentage of monocytes (×10^9^/L, %)	4.05(3.00, 0.5.73)	4.00(3.08, 5.73)	4.35(2.83, 5.95)	−0.260	0.795	4.00(2.98, 5.85)	4.35(3.00, 5.57)	−0.534	0.593
ST group	57(58.2%)	41(68.3%)	16(42.1%)	6.577	0.010*	34(70.8%)	23(46.0%)	6.207	0.013*
SMIS	29(50.9%)	21(51.2%)	8(50.0%)			20(58.8%)	9(39.1%)		
CBDS	28(49.1%)	20(48.8%)	8(50.0%)	0.007	0.934	14(41.2%)	14(60.9%)	2.129	0.145

CT, Computed tomography; GCS, Glasgow Coma Scale; IQR, Interquartile range; mRS, Modified Rankin Scale; NIHSS, National Institutes of Health Stroke Scale; and ▲ = ^−^x ± s. *Significant variables at the *P* < 0.05 level.

## 3 Results

### 3.1 Participants

**Figure 4:**
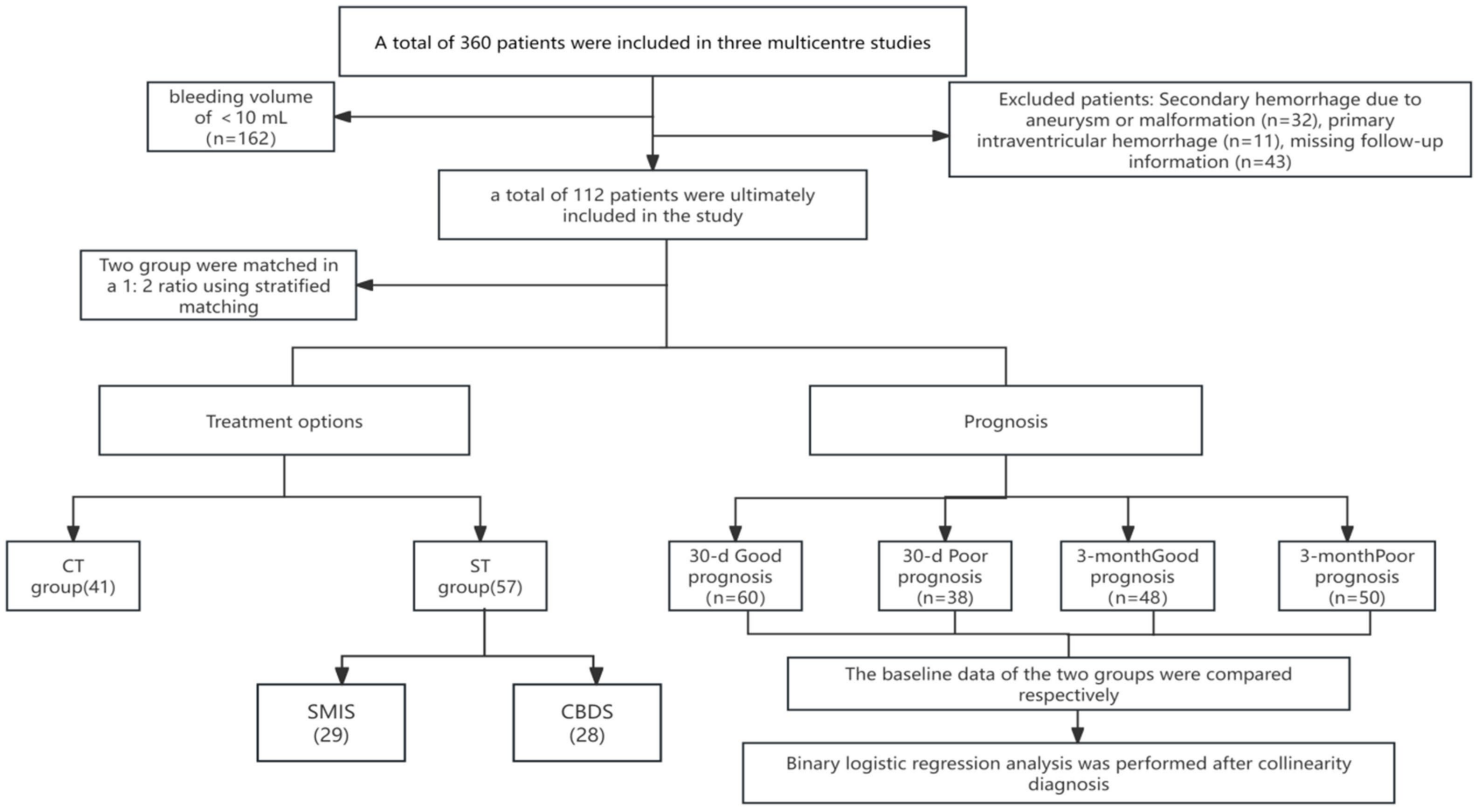
Study flowchart.

A total of 112 SCH patients were included in the study, and after using a 1:2 propensity score matching, there were 57 patients in the CT group and 41 patients in the ST group. Comparing the baseline data of two groups of patients, it was found that 98 SCH patients could be further divided into a 30 day good prognosis group (*n* = 60) and a 30 day poor prognosis group (*n* = 38), a 3-month good prognosis group (*n* = 48), and a 3-month poor prognosis group (*n* = 50). After excluding collinearity factors through collinearity diagnosis, a binary logistic regression model was used to analyze the independent correlation between good prognosis and poor prognosis (Figure 4).

### 3.2 Baseline comparison of the two treatment methods

The CT and ST groups included 41 and 57 patients, respectively, with no significant differences in sex, age, smoking and Alcohol consumption, diabetes history, systolic and diastolic blood pressure upon admission, NIHSS and GCS scores, maximum hematoma diameter, obstructive hydrocephalus, hematoma expansion, secondary epilepsy upon discharge, pulmonary complications, cardiac complications, and posterior cranial fossa constriction syndrome between the two groups (*p* > 0.05). However, the ST group showed a higher proportion of favorable 30-d prognosis and 3-month prognosis [41(71.9%) vs. 19(46.3%), *p* = 0.010], [34(59.6%) vs. 14(34.1%), *p* = 0.013], and the mortality rate was lower than that of CT group [10(17.9%) vs. 15(39.5%), *p* = 0.020], Our study also found that the ST group had higher blood pressure, Leukocyte than the CT group [46(86.0%) vs. 27(65.9%), *p* = 0.019], [12.89(10.39, 16.10) vs. 10.43(8.34, 12.68), *p* = 0.008], as shown in Table 1.

### 3.3 Baseline comparison of the 30-d prognosis and 3-month patient prognosis

A total of 98 patients with SCH were included in the study, of whom 60 had a favorable prognosis at 30 days and 38 had an unfavorable prognosis. At 3 months, 48 patients showed a favorable prognosis and 50 showed an unfavorable prognosis. Whether it is a 30-d prognosis or a 3-month prognosis, The two groups showed no statistical differences in terms of sex, age, smoking and alcohol consumption, history of hypertension, diabetes, systolic blood pressure, diastolic blood pressure, Hematoma volume, or maximum hematoma diameter (*p* > 0.05).

However, univariate analysis showed that the proportion of patients with good prognosis at 30-d and 3-month in the ST group was higher than that of patients with poor prognosis [41(68.3%) vs. 16(42.1%), *p* = 0.010, 34(70.8%) vs. 23(46.0%), *p* = 0.013]. Additionally, the proportion of patients with posterior fossa compression syndrome in the group with good prognosis at 30 days and 3 months was lower than that in the group with poor prognosis at 30 days and 3 months [15(25.0%) vs. 19(50.0%), *p* = 0.011], [12(25.0%) vs. 22(44.0%), *p* = 0.048]. The proportion of patients with death in the group with good prognosis at 30 days and 3 months was lower than that in the group with poor prognosis at 30 days and 3 months [5(8.5%) vs. 20(57.1%), *p* = 0.001], [3(6.4%) vs. 22(46.8%), *p* = 0.001], with significant differences observed in NIHSS and GCS scores. Our univariate analysis also showed that the platelet count of patients with good prognosis was higher than that of patients with poor prognosis at 30 days and 3 months (199.83 ± 60.48 vs.172.02 ± 60.69, *p* = 0.037), (202.80 ± 60.36 vs. 176.12 ± 60.83, *p* = 0.035). In our univariate analysis of one-month prognosis, there were significant differences in the length of hospital stay and cerebral hernia (*p* < 0.05) (Table 2).

Among the 57 patients who underwent surgical treatment, further analysis showed that 29 underwent SMIS and 28 CBDS. The good prognosis rates at 1 month and 3 months for SMIS and CBDS group were 51.2 and 58.8%, 48.8 and 41.2%, respectively, with no statistically significant difference between the groups (*p* > 0.05).

After excluding collinear variables with VIF values > 3 in the collinearity diagnosis (Tables 3, 4), these variables were ultimately included in the binary logistic regression model.

**Table 3:** Thirty-day prognosis Collinearity diagnosis.

Characteristic	*t*	*P*	Tolerance	VIF
Hospitalization days	−0.019	0.985	0.968	1.033
NIHSS	−1.662	0.100	0.264	3.789
GCS	1.142	0.256	0.261	3.824
Pulmonary complications	−2.210	0.029	0.972	1.029
Tight posterior fossa	−1.713	0.090	0.961	1.041
ST group	2.547	0.012	0.972	1.028

**Table 4:** Three-month prognosis Collinearity diagnosis.

Characteristic	*t*	*P*	Tolerance	VIF
NIHSS	2.439	0.017	0.428	2.336
GCS	−0.601	0.549	0.436	2.293
Tight posterior fossa	−1.094	0.277	0.965	1.036
Platelet	−2.276	0.025	0.965	1.036
ST group	2.739	0.007	0.982	1.018

The final binary logistic regression model identified surgical treatment [odds ratio (OR): 4.898, 95% CI:1.559–15.388, *p* = 0.007], (OR: 3.965, 95% CI:1.429–11.004, *p* = 0.008), Platelet (OR: 0.989, 95% CI:0.980–0.998, *p* = 0.021), (OR: 0.990, 95% CI:0.982–0.999, *p* = 0.027), and NIHSS (OR: 1.073, 95% CI: 1.016–1.132, *p* = 0.011), (OR: 1.068, 95% CI: 1.011–1.128, *p* = 0.019) as independent predictors of 30-d prognosis and 3-month prognosis in patients with SCH (Tables 5, 6).

**Table 5:** Multivariable binary logistic regression analysis of factors associated with favorable 30-d prognosis prognosis.

Variables	OR	95% CI	*p*
Hospitalization days	0.993	0.966–1.021	0.625
Cerebral hernia	0.140	0.015–1.264	0.080
Tight posterior fossa	0.478	0.165–1.380	0.172
Platelet	0.989	0.980–0.998	0.021
NIHSS	1.073	1.016–1.132	0.011
GCS	0.974	1.016–1.132	0.763
ST group	4.898	1.559–15.388	0.007

**Table 6:** Multivariable binary logistic regression analysis of factors associated with favorable 3-month prognosis prognosis.

Variables	OR	95% CI	*p*
NIHSS	1.068	1.011–1.128	0.019
GCS	0.938	0.796–1.104	0.440
Platelet	0.990	0.982–0.999	0.027
Tight posterior fossa	0.637	0.229–1.770	0.387
ST group	3.965	1.429–11.004	0.008

## 4 Discussion

In our study, we found which includes only Patients with SCH hematoma was >10 mL, a higher proportion of patients in the ST group had a good prognosis at 30-d prognosis and 3-month prognosis than those in the CT group [41(68.3%) vs. 16(42.1%), *p* = 0.010], [34(70.8%) vs. 23(46.0%), *p* = 0.013]. Moreover, the results showed that surgical treatment was an independent predictor of 30-d prognosis and 3-month prognosis (OR: 4.898, 95% CI: 1.559–15.388, *p* = 0.007, OR: 3.965, 95% CI: 1.429–11.004, *p* = 0.008). Considering that cerebellar hemorrhage can easily compress the brainstem and fourth ventricle (24), our research suggests that for patients with hematomas exceeding 10 mL, adopting an active surgical intervention strategy may bring clinical benefits. However, this conclusion still needs further validation through prospective studies.

The ICH Score is a clinical grading scale composed of factors related to a basic neurological examination, a baseline patient characteristic, and initial neuroimaging. It is recognized that there is a risk of rapid clinical deterioration caused by brainstem compression or obstructive hydrocephalus during SCH. Consequently, patients with cerebellar bleeds who present with a high ICH score have been consistently shown to have a substantially higher 30-day mortality compared to patients with supratentorial hemorrhages of a similar score (25–27). Recent years have seen a significant increase in research focusing on infratentorial intracerebral hemorrhage, particularly in relation to the posterior cranial fossa. Given the anatomical complexity of this region, it is imperative to give particular consideration to the posterior cranial fossa for hemorrhage when it occurs in the cerebellum (28). ICH scores is an effective tool for predicting the survival rate and prognosis of patients with SCH. In cases of severe SCH, with the ICH scores than 3, the administration of surgical treatment may be of benefit (14). This finding is highly consistent with our research, and we recommend that immediate surgical intervention be performed to remove the hematoma and improve patient prognosis when cerebellar hemorrhage exceeds 10 mL.

The findings of some retrospective studies are consistent, suggesting that regardless of age, hematoma size, or consciousness status, patients with SCH should undergo suboccipital bone flap decompression as early as possible to remove the hematoma (6) and restore their neurological function as soon as possible. In a single-center retrospective study of 85 patients with SCH conducted in 2017, the hematoma clearance rate of the suboccipital bone flap decompression group was significantly higher than that of the conservative treatment (CT) group (*p* < 0.001), with the former showing better neurological functional prognosis than the latter (7) (*p* < 0.004). However, another a systematic review study found that CT was associated with a better prognosis than ST for patients with SCH (29). Unfortunately, due to the significant differences in characteristics between patients who underwent CT and ST and the high variability of treatment indications, meaningful comparisons of results could not be performed. It has been demonstrated that surgical removal of hematomas does not enhance the functional prognosis of patients with SCH compared with CT (12).

In 2022, the American Stroke Association recommended emergency surgical removal of hematoma for patients with cerebellar hemorrhage ≥ 15 mL or worsening neurological function to reduce further deterioration (4). Meanwhile, Al-Kawaz et al. found a significant correlation between the patient survival rate and surgical removal of the hematoma (5). Other studies have suggested that patients with SCH who experience brainstem compression, fourth ventricle obstruction, cerebral pool obstruction, hydrocephalus, and progressive deterioration of consciousness should undergo immediate hematoma evacuation surgery to improve patient prognosis (7). The European Stroke Organization also believes that combined SCH and hydrocephalus treatment is effective for intraventricular drainage. In cases of severe cerebellar hemorrhage presenting with signs of impending transtentorial herniation or obstructive hydrocephalus, emergency external ventricular drainage (EVD) was considered as a life-saving procedure to rapidly lower intracranial pressure (30). It is important to note that EVD is generally effective in relieving sudden increases in intracranial pressure prior to performing a definitive suboccipital craniotomy. Nonetheless, owing to the pressure gradient established between the supratentorial and infratentorial compartments, fluctuations in pressure have been demonstrated to heighten the probability of an upward hernia (31). However, based on our research, we recommend surgical treatment for patients when the hematoma volume is >10 mL. And in our surgical subgroup analysis, due to limited sample size, it is not yet possible to draw definitive conclusions about the advantages and disadvantages of the two surgical methods. Prospective studies are needed in the future to specifically compare the effectiveness of different surgical techniques.

In our study, we found that NIHSS score were independent predictors of 30-d prognosis and 3-month prognosis in patients with SCH (OR: 1.073, 95% Cl: 1.016–1.132, *p* = 0.011, OR:1.068, 95% Cl:1.011–1.128, *p* = 0.019), which is consistent with some research findings that NIHSS score is an independent predictor of short-term prognosis for patients (3). We also identified that platelet count serves as an independent predictor of the short-term prognosis for patients (OR: 0.989, 95% Cl: 0.980–0.998, *p* = 0.021, OR: 0.990, 95% Cl: 0.982–0.999, *p* = 0.027). This study identified that a higher platelet count serves as an independent predictor of favorable short-term functional outcomes in patients. Specifically, platelets play a crucial role in mitigating hematoma expansion through their fundamental hemostatic functions (32, 33). In addition, there is an increasing body of evidence suggesting that platelets possess immunomodulatory and neuroprotective properties. These characteristics may be particularly significant in alleviating secondary inflammatory damage following cerebellar hemorrhage (34).

In recent years, the management of ICH has increasingly emphasized comprehensiveness alongside systematic approaches. As highlighted in the recently published Code ICH international consensus, multidisciplinary assessment and rapid intervention during the acute phase of ICH are crucial for improving patient outcomes. This consensus specifically underscores the value of clear treatment goals and timely decision-making in saving critically ill patients (35). Our study focuses on cerebellar hemorrhage, an ICH subtype that often requires urgent neurosurgical intervention due to its unique anatomical location. Our research found that when cerebellar hemorrhage exceeds 10 mL, surgical removal of hematoma is significantly correlated with patient prognosis. This association holds true regardless of whether open craniotomy or minimally invasive surgery is performed, which aligns with the Code ICH consensus’s advocacy for “aggressive measures in the acute phase.” Integrating our findings into the comprehensive management pathway outlined by Code ICH may further optimize acute-phase management for cerebellar hemorrhage patients, ultimately improving their survival rates and neurological recovery.

Although our study employed a retrospective design, it may still have some potential biases, including unmeasured confounding factors and incomplete medical records. Second, our study had a relatively small sample size, which may limit its statistical power. Future well-designed, large-sample multicenter prospective studies or randomized controlled trials are needed to provide conclusive evidence of the observed trends. Another limitation of this study is that it mainly relies on the mRS as a global functional indicator, and fails to include standardized neurological assessments of cerebellar functional characteristics such as coordination function, articulation disorders, and balance ability. Although mRS has good clinical practicality and comparability, it may not be sensitive enough to subtle changes in cerebellar specific defects. We speculate on the potential impact of two treatment strategies on these secondary outcomes: (1) Surgical treatment may theoretically provide a more favorable anatomical environment for the recovery of motor coordination and balance function by rapidly relieving the compression of deep cerebellar nuclei and brainstem caused by hematoma (36). (2) Conservative treatment may achieve good functional outcomes for patients with small hematomas and clear consciousness through natural recovery and rehabilitation training, but persistent hematoma compression may increase the risk of chronic ataxia or articulation disorders (12, 37). Future research urgently needs to integrate specific scales such as the International Cooperative Ataxia Rating Scale and Berg Balance Scale to accurately quantify the impact of treatment on cerebellar function.

## 5 Conclusion

Surgical treatment is an independent predictor of good 30-d prognosis and 3-month prognosis for SCH patients, therefore we recommend that SCH patients also undergo surgical treatment when the bleeding volume is greater than 10 mL. These findings also indicate that in terms of short-term prognosis, surgical treatment has better prognosis and lower mortality rate for patients with hematoma volume>10 mL compared to conservative treatment.

## Data Availability

The original contributions presented in the study are included in the article/supplementary material, further inquiries can be directed to the corresponding author.
